# Blast-Induced Neurotrauma Models and Their Requirements

**DOI:** 10.3389/fneur.2014.00128

**Published:** 2014-07-10

**Authors:** Ibolja Cernak

**Affiliations:** ^1^Military and Veterans’ Clinical Rehabilitation Research, University of Alberta, Edmonton, AL, Canada

**Keywords:** blast, blast-induced neurotrauma, models, animal models of traumatic brain injury, computer models

Although advances are being made in identifying some of the essential mechanisms that may lead to chronic neurological deficits after blast-exposure(s), clinical needs continue to exceed current knowledge. In their current article, Brenner and colleagues address the importance of basic science in guiding new diagnostic and treatment strategies. Together with Wilkinson et al., they emphasize one of the basic tenets of experimental biomedical research: to reliably reproduce pathological conditions including neuroendocrine (1) and behavioral changes (2) seen in blast-exposed soldiers.

The existing literature on the pathobiology of blast-induced neurotrauma (BINT) is often contradictory, in part, due to the lack of understanding of the physics of blast, and a broad range of experimental animals and models being used (3). Moreover, as Risling and Davidsson (4) pointed out, the experimental modeling of the BINT is especially challenging due to deficient exposure data from actual operational/clinical situations.

It is noteworthy to remind the researchers that the purpose of experimental models of traumatic brain injury (TBI), thus BINT, is to replicate certain pathological components or phases of clinical trauma in experimental animals aiming to address pathology and/or treatment. Hence, the design and choice of the chosen specific model should emulate the goal of research (5). Regardless of the research questions to be addressed, the criteria every clinically and militarily relevant BINT model should fulfill are the following: (1) the injurious component of the blast should be clearly identified and reproduced in controlled, reproducible, and quantifiable manner; (2) the inflicted injury should be reproducible, quantifiable, and mimic components of human BINT; (3) the injury outcome established based on morphological, physiological, biochemical, and/or behavioral parameters should be related to the chosen injurious component of the blast; and (4) the mechanical properties (intensity, complexity of blast signature, and/or its duration) of the injurious factor should predict the outcome severity.

Based on the research question, the researcher should clearly define the blast effects to be reproduced. If the choice is primary blast, the animal’s body must be fixed to prevent blast-induced acceleration of the body/head during the exposure. Namely, if the body/head is allowed to move, the injury mechanisms would involve both primary and tertiary blast effects, which would make the interpretation of the results complicated. Next, the biological complexity of the research study should be established; this will dictate the choice of research environment, methods of generating a shock wave, research subjects and their positioning, and length of the experiment. The article published by Ahlers and colleagues (6) demonstrates the importance of a well-defined experimental setting including the animal’s body position toward incoming shock wave, among others, for the outcome of the experiments, thus for the final conclusion the study will imply.

Based on well-formulated research question and identified scale of complexity, the researcher should choose among non-biological (*in silico* or surrogate physical) or biological (*in vitro, ex vivo*, or *in vivo*) models that would suit the task. Zhang et al. (7) performed an *in silico* finite element (FE) study to evaluate the human head response against blast loadings with and without Advanced Combat Helmet. Effgen et al. (8) describe an *in vitro* model using organotypic hippocampal slice cultures, exposed to overpressure conditions that are generated by a compressed-gas shock tube. Many *in vivo* TBI and BINT models use rodents due to ethical, technical, and/or financial limitations linked to experimental studies using phylogenetically higher species. However, it has been suggested that the rodent lissencephalic cortex makes mice and rats inappropriate for modeling the more complex injury-induced changes in cognition and behavior. Shridharani and colleagues (9) developed a model using porcine subjects exposed to primary blast overpressures that are generated using a compressed-gas shock tube; their article evaluates the mechanical response of pig’s head, i.e., a head of a large animal with similar body mass to a human, to primary blast waves. The authors concluded that when developing an animal BINT model, the scaling laws for adjusting a scenario of a human blast-exposure to experimental species should consider morphologic differences between species as additional factors beyond body- or head-mass. Reliable experimental models of BINT are of vital importance not only in the identification of the complex mechanisms leading to long-term functional deficits, but also in guiding novel approaches to diagnosis and treatment modalities. Svetlov and colleagues (10) used a rat model of blast-exposure to generate some of the hallmarks of BINT and identify a set of potential biomarkers to measure the onset and progress of blast-induced neurological deficits.

There is a dire need for a well-coordinated, multidisciplinary research approach to the problem of blast injuries, including BINT. Our tasks ahead are numerous: to define the injury tolerance levels; develop reliable, militarily, and clinically relevant experimental models; and define the injury mechanisms underlying acute and chronic consequences of blast-exposure(s). These challenging tasks can only be achieved with a unified front of physicists, military scientists, biomedical researchers, and clinicians who use out-of-the-box thinking and novel research approaches.

## Conflict of Interest Statement

The author declares that the research was conducted in the absence of any commercial or financial relationships that could be construed as a potential conflict of interest.

## References

[1] Wilkinson CW Pagulayan KF Petrie EC Mayer CL Colasurdo EA Shofer JB High prevalence of chronic pituitary and target-organ hormone abnormalities after blast-related mild traumatic brain injury. Front Neurol (2012) 3:11 10.3389/fneur.2012.00011 22347210 PMC3273706

[2] Brenner LA Bahraini N Hernandez TD Perspectives on creating clinically relevant blast models for mild traumatic brain injury and post traumatic stress disorder symptoms. Front Neurol (2012) 3:31 10.3389/fneur.2012.00031 22408635 PMC3296058

[3] Cernak I Noble-Haeusslein LJ Traumatic brain injury: an overview of pathobiology with emphasis on military populations. J Cereb Blood Flow Metab (2010) 30(2):255–66 10.1038/jcbfm.2009.203 19809467 PMC2855235

[4] Risling M Davidsson J Experimental animal models for studies on the mechanisms of blast-induced neurotrauma. Front Neurol (2012) 3:30 10.3389/fneur.2012.00030 22485104 PMC3317041

[5] Cernak I Animal models of head trauma. NeuroRx (2005) 2(3):410–22 10.1602/neurorx.2.3.410 16389305 PMC1144485

[6] Ahlers ST Vasserman-Stokes E Shaughness MC Hall AA Shear DA Chavko M Assessment of the effects of acute and repeated exposure to blast overpressure in rodents: toward a greater understanding of blast and the potential ramifications for injury in humans exposed to blast. Front Neurol (2012) 3:32 10.3389/fneur.2012.00032 22403572 PMC3293241

[7] Zhang L Makwana R Sharma S Brain response to primary blast wave using validated finite element models of human head and advanced combat helmet. Front Neurol (2013) 2:4 10.3389/fneur.2013.00088 23935591 PMC3731672

[8] Effgen GB Hue CD Vogel E III Panzer MB Meaney DF Bass CR A multiscale approach to blast neurotrauma modeling: part II: Methodology for inducing blast injury to in vitro models. Front Neurol (2012) 3:23 10.3389/fneur.2012.00023 22375134 PMC3285773

[9] Shridharani JK Wood GW Panzer MB Capehart BP Nyein MK Radovitzky RA Porcine head response to blast. Front Neurol (2012) 3:70 10.3389/fneur.2012.00070 22586417 PMC3347090

[10] Svetlov SI Prima V Glushakova O Svetlov A Kirk DR Gutierrez H Neuro-glial and systemic mechanisms of pathological responses in rat models of primary blast overpressure compared to “composite” blast. Front Neurol (2012) 3:15 10.3389/fneur.2012.00015 22403567 PMC3275793

